# Transcriptomic and functional analyses reveal the molecular mechanisms underlying Fe-mediated tobacco resistance to potato virus Y infection

**DOI:** 10.3389/fpls.2023.1163679

**Published:** 2023-03-30

**Authors:** Chuantao Xu, Huiyan Guo, Rui Li, Xinyu Lan, Yonghui Zhang, Qiang Xie, Di Zhu, Qing Mu, Zhiping Wang, Mengnan An, Zihao Xia, Yuanhua Wu

**Affiliations:** ^1^ Liaoning Key Laboratory of Plant Pathology, College of Plant Protection, Shenyang Agricultural University, Shenyang, China; ^2^ Luzhou City Company of Sichuan Province Tobacco Company, Luzhou, China; ^3^ Guizhou Qianxinan Prefectural Tobacco Company, Xingyi, China

**Keywords:** PVY, Fe, full-length transcriptome, Illumina RNA sequencing, virus-induced gene silencing

## Abstract

Potato virus Y (PVY) mainly infects Solanaceous crops, resulting in considerable losses in the yield and quality. Iron (Fe) is involved in various biological processes in plants, but its roles in resistance to PVY infection has not been reported. In this study, foliar application of Fe could effectively inhibit early infection of PVY, and a full-length transcriptome and Illumina RNA sequencing was performed to investigate its modes of action in PVY-infected *Nicotiana tabacum*. The results showed that 18,074 alternative splicing variants, 3,654 fusion transcripts, 3,086 long non-coding RNAs and 14,403 differentially expressed genes (DEGs) were identified. Specifically, Fe application down-regulated the expression levels of the DEGs related to phospholipid hydrolysis, phospholipid signal, cell wall biosynthesis, transcription factors (TFs) and photosystem I composition, while those involved with photosynthetic electron transport chain (PETC) were up-regulated at 1 day post inoculation (dpi). At 3 dpi, these DEGs related to photosystem II composition, PETC, molecular chaperones, protein degradation and some TFs were up-regulated, while those associated with light-harvesting, phospholipid hydrolysis, cell wall biosynthesis were down-regulated. At 9 dpi, Fe application had little effects on resistance to PVY infection and transcript profiles. Functional analysis of these potentially critical DEGs was thereafter performed using virus-induced gene silencing approaches and the results showed that *NbCat-6A* positively regulates PVY infection, while the reduced expressions of *NbWRKY26*, *NbnsLTP*, *NbFAD3* and *NbHSP90* significantly promote PVY infection in *N. benthamiana*. Our results elucidated the regulatory network of Fe-mediated resistance to PVY infection in plants, and the functional candidate genes also provide important theoretical bases to further improve host resistance against PVY infection.

## 1 Introduction

Potato virus Y (PVY) is the typical member of the genus *Potyvirus* in the family *Potyviridae* (Rybicki, 2015). PVY infects a wide host range mainly within the Solanaceous crops, such as potato, tomato, pepper and tobacco, and causes considerably economic losses worldwide (Gibbs et al., 2020; Yang et al., 2021). As known, viruses often rely on a complex network of interactions with host proteins to promote infection in plants (Garcia-Ruiz, 2019). In the arms race between hosts and pathogens, plants have evolved a series of strategies to resist viral infections (Lebaron et al., 2016). Virus infections also alter the expression levels of a large number of host genes (Garcia-Ruiz, 2019). A study has shown that the expression levels of differentially expressed genes (DEGs) related to plant-pathogen interactions, including heat shock proteins, are down-regulated, while those of terpene synthase and protein kinase encoding genes are up-regulated in the susceptible potato varieties after PVY infection (Ross et al., 2022). In PVY-infected tobacco plants, DEGs related to DNA/RNA binding, catalytic activity and signaling molecules are significantly enriched, and PVY-derived siRNAs are shown to target translationally controlled tumor protein (*NtTCTP*) mRNA that is associated with host resistance to viral infection (Guo et al., 2017).

Microelements, such as boron (B), chlorine (Cl), copper (Cu), iron (Fe), manganese (Mn) and zinc (Zn), play essential roles during plant growth (Merchant, 2010). Among them, Fe is known as the third most limiting microelements for plants due to its low solubility in alkaline and calcareous soils (Gyana and Rout, 2015). Fe is required for a variety of biological processes, such as DNA synthesis, photosynthesis, respiration, nitrogen reduction, biosynthesis and repair of nucleotide, amino acids, proteins, cofactors, and vitamins (Chhabra et al., 2020; Hendrix et al., 2022). Fe deficiency causes up-regulation of *bHLH38*, *bHLH100* and *bHLH101*, and inhibits flowering in *Arabidopsis* (Chen et al., 2021). The expression levels of the basic helix-loop-helix transcription factor *FER*, the ferric-chelate reductase *LeFRO1* and the Fe (II) transporter *LeIRT1* genes are up-regulated in the iron-deficient conditions, which promotes the production of nitric oxide (NO) in tomato plants (Graziano and Lamattina, 2007). In *Arabidopsis*, high iron concentrations promote NO production and induce *AtFer1* and *AtFer4* expression, thus allowing excess iron to be preserved in a bioavailable and non-toxic form (Arnaud et al., 2006). In addition, the regulatory roles of Fe in plant resistance during pathogen infection have been well investigated. Foliar spraying of Fe_3_O_4_ nanoparticles can activate the oxidative stress response, induce the synthesis of salicylic acid (SA), and up-regulate the expression of pathogenesis-related (PR) proteins, thus enhancing the resistance of plants to the infection of tobacco mosaic virus (TMV) (Cai et al., 2020). It was also indicated that iron treatment can induce reactive oxygen species (ROS) burst to regulate the resistance to *Curvularia lunata* in maize (Fu et al., 2022).

Next-generation sequencing (NGS) technology based on Illumina platform is a powerful method to provide insights into mechanisms underlying processes of global gene expression and secondary metabolism (Unamba et al., 2015). The single molecule real-time (SMRT) sequencing based on Pacific BioSciences (PacBio) platform allows the direct reading of cDNA and the maximum reading length to 70 kb, and accurately reconstructs full-length splice variants (Korlach et al., 2017). Overall, combining analysis of NGS and SMRT sequencing can provide high-quality, accurate, and complete isoforms in transcriptome studies, which is thereby conducive to discovering more alternative splicing (AS) isoforms, long non-coding RNAs (lncRNAs) and fusion genes. A reference transcriptome of impatiens infected with downy mildew has been constructed by full-length transcriptome sequencing and RNA sequencing (RNA-Seq), which provided a comprehensive data source to screen resistance genes (Peng et al., 2021).

Investigations on the roles of trace elements in plant antiviral responses are still limited. Our previous studies have shown that boron can inhibit cucumber green mottle mosaic virus (CGMMV) infection in watermelon by regulating the expression levels of genes related to carbohydrate metabolism, hormone biosynthesis, cell wall catabolism and ROS burst (Bi et al., 2022a; Bi et al., 2022b; Guo et al., 2022). In this study, we demonstrated that foliar spraying of Fe inhibited PVY infection in *N. tabacum*, and analyzed the expression patterns of tobacco genes under four different treatments with or without PVY infection after spraying Fe or H_2_O at 1, 3, 9 days post inoculation (dpi) by full-length transcriptome and RNA-Seq analyses. The results revealed that PVY infection mainly affected the expression levels of host genes related to photosynthesis and biosynthesis, while Fe treatment regulated genes associated with lipid metabolism, photosynthesis, endoplasmic reticulum protein processing and cell wall biogenesis. Moreover, the roles of several genes were characterized by down-regulated their expressions through virus-induced gene silencing (VIGS) assays in the PVY-infected *N. benthamiana* plants. These results provide a new method for prevention of PVY infection and lay a theoretical foundation for disease resistant breeding in Solanaceous crops.

## 2 Materials and methods

### 2.1 Plant growth and virus inoculation

*N. tabacum* L. cv. K326 and *N. benthamiana* plants were cultivated in the artificial climate chamber with a day/night temperature of 25°C, 65 ± 5% relative humidity, and 16 h/8 h light/dark cycle environmental conditions. Potato virus Y (PVY-LN, GenBank ID: JQ971975) was isolated and purified by our laboratory and propagated on tobacco. *N. tabacum* leaves were sprayed twice every 2 days with H_2_O or Fe^2+^ (EDTA-Fe) or MgSO_4_ or CuSO_4_ with concentration of 3.36 mg·L^-1^ or 240 mg·L^-1^ or 0.8 mg·L^-1^ at 4-5 leaf stage and then inoculated with phosphate-buffered saline (PBS solution) or PVY after one day, respectively (PBS solution + H_2_O, P + H; PBS solution + Fe, P + Fe; PBS solution + Mg, P + Mg; PBS solution + Cu, P + Cu; PVY + H_2_O, PVY + H; PVY + Fe; PVY + Mg; PVY + Cu). The samples were harvested from inoculated leaves at 1 dpi, and systematic leaves at 3 dpi and 9 dpi, and three independent experiments were performed in this study.

### 2.2 Library preparation and SMRT sequencing

We mixed all samples of different treatments (P + H, P + Fe, P + Mg, P + Cu, PVY + H, PVY + Fe, PVY + Mg, PVY + Cu) at 3 time points into one sample for full-length transcriptome analysis based on SMRT sequencing. Total RNA was extracted using a Trizol reagent (Invitrogen, CA, USA). The RNA quantity and purity were analyzed by Bioanalyzer 2100 and RNA 1000 Nano LabChip Kit (Agilent, CA, USA) with RIN number > 7.0. Full-length cDNA was synthesized using a SMAR Ter™ PCR cDNA Synthesis Kit (Clontech, CA, USA). The generated cDNA was then re-amplified using PCR. After end repair, the SMRT adaptor with a hairpin loop structure was ligated to the cDNA. The cDNA library was then constructed *via* exonuclease digesting. After quality measurement of the cDNA library, SMRT sequencing was performed using the Pacific Bioscience Sequel platform (Biomarker Technologies Co. Ltd., Beijing, China). The reference genome of tobacco K326 (https://solgenomics.net/organism/Nicotiana_tabacum/genome) was used. The sequencing data were deposited in the SRA database at NCBI with the accession number PRJNA903693.

### 2.3 Illumina RNA-seq library construction and sequencing

The mRNA extracted from samples in four different treatments (P + H, P + Fe, PVY + H, PVY + Fe) was purified using oligo (dT)-attached magnetic beads. Fragmentation was conducted in the NEBNext First Strand Synthesis Reaction Buffer. First-strand cDNA was obtained based on the random hexamers, and then the second-strand cDNA was synthesized with dNTPs, RNase H, and PrimeStar GXL DNA polymerase. The synthesized cDNA was purified with AMPure XP beads. After end repairing, adding poly-A and adaptor ligation, AMPure XP beads were used for size selection. The generated cDNA was then amplified for construction of cDNA libraries. The qualified libraries were pair-end sequenced on the Illumina HiSeq TM2500 (Biomarker Technologies Co. Ltd., Beijing, China). The sequencing data were deposited in the SRA database at NCBI with the accession number PRJNA903693. The relative gene expression levels were normalized as fragments per kilobase of transcript per million mapped reads (FPKM). The genes with threshold of false discovery rate (FDR) < 0.05 and |log2 fold change| ≥ 1 were defined as DEGs.

### 2.4 Functional annotation of transcripts

Gene Ontology (GO) enrichment of DEGs were analyzed by a GOseq R packages based Wallenius non-central hyper-geometric distribution (Young et al., 2010). A KOBAS software was used to perform KEGG enrichment analyses of DEGs (Mao et al., 2005).

### 2.5 WGCNA analyses

Weighted gene co-expression network analysis (WGCNA) was used to construct gene co-expression networks. Highly co-expressed gene modules were obtained using the WGCNA v3.1.1 package in R language (Langfelder et al., 2009). A gene expression adjacency matrix was constructed to analyze the network topology with minModuleSize of 30, and minimum height for merging modules of 0.1262.

### 2.6 Virus-induced gene silencing assays

The homologs of five DEGs were selected for functional verification using a tobacco rattle virus (TRV)-based VIGS vector in *N. benthamiana*, respectively. These fragments were amplified using specific primers (**Supplementary Table 1**) and PrimeSTAR^®^ Max DNA Polymerase (TaKaRa, Dalian, China) through PCR. The details of construction of TRV-based VIGS vectors and infiltration of *Agrobacterium tumefaciens* referred to our previous study (Guo et al., 2022). After 10 days post infiltration, the upper non-infiltrated leaves were mechanically inoculated with PVY crude extracts. After 10 dpi, we monitored *N. benthamiana* plants symptoms and collected the upper two leaves to measure gene silencing efficiency and PVY accumulation.

### 2.7 Western blot analyses

Total proteins from tobacco K326 inoculated leaves at 1 dpi and systematic leaves at 3, 5, 7, 9 dpi, as well as the upper two leaves of *N. benthamiana* plants were extracted using a Plant Protein Extraction Kit (Solarbio, Shanghai, China) and quantified using the BCA protein quantification kit (Beyotime, Shanghai, China), respectively. After the completion of electrophoresis, the proteins separated by 12% SDS-PAGE were transferred to 0.20 μm polyvinylidene fluoride (PVDF) membranes (Millipore, Billerica, USA), which were then incubated in blocking solution for one hour (Solarbio, Shanghai, China). PVY CP monoclonal antibody was used at dilution of 1: 1000 (Youlong, Shanghai, China). Beta-actin antibody was used at dilution of 1: 5000 (Proteintech, Chicago, USA). After incubation with primary and secondary antibodies (ABclonal, Wuhan, China), membranes were washed twice for 10 min with 1×TTBS, and then transferred into ECL solution (Millipore, Billerica, USA) to detect signals by Tanon Chemiluminescence Gel Imager (Tanon, Shanghai, China).

### 2.8 Reverse transcription-quantitative real-time PCR (RT-qPCR)

The RNA of the inoculated leaves at 1 dpi and systematic leaves at 3, 5, 7, 9 dpi from *N. tabacum* K326, as well as the upper two leaves of *N. benthamiana* plants was extracted by TRIzol reagent (Tiangen, Beijing, China) and was reverse transcribed to yield cDNA with a HiScript II Q Select RT SuperMix for qPCR (gDNA eraser) kit (Vazyme, Nanjing, China). The primer sets for partial sequence amplification were designed within the CDS region of nucleotide sequences (**Supplementary Table 1**). RT-qPCR analyses were performed using ChamQ Universal SYBR qPCR Master Mix (Vazyme, Nanjing, China) on a StepOne Plus real-time PCR system (ThermoFisher, Waltham, USA). The relative expression levels of genes were assessed by the 2^−ΔΔCT^ method with the normalization using *Ntubc2* (AB026056.1) in *N. tabacum* and *NbActin* (AY179605.1) in *N. benthamiana* as reference genes by three biological replicates, respectively.

### 2.9 Statistical analyses

The data were presented as mean values ± standard error of three biological replicates and analyzed using SPSS Version 25.0 (IBM Inc., Armonk, USA). The differences among groups were analyzed through two-tailed *t* test and one-way analysis of variance (Duncan).

## 3 Results

### 3.1 Foliar application of exogenous Fe could alleviate PVY infection in tobacco plants

**Figure 1:**
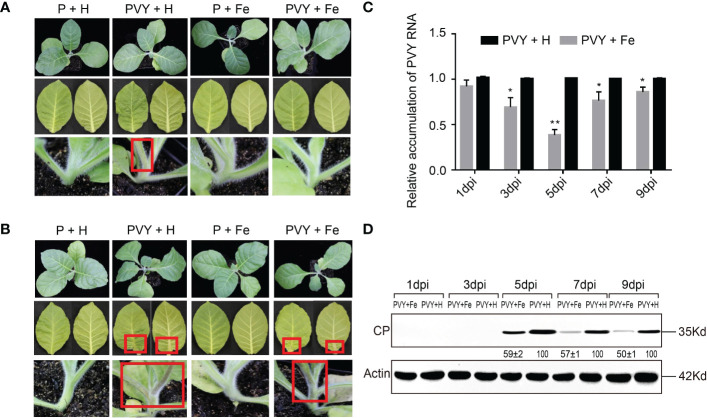
Foliar application of exogenous Fe alleviated PVY infection in tobacco plants. **(A)** Symptoms of the whole plant, leaves and stems at 7 dpi in four treatments. **(B)** Symptoms of the whole plant, leaves and stems at 9 dpi in four treatments. **(C)** The accumulation of PVY genomic RNA in tobacco leaves of PVY + H and PVY + Fe groups determined by RT-qPCR at 1, 3, 5, 7, and 9 dpi. Asterisks indicate statistically significant differences compared to control (Student’s *t*-test): *P < 0.05, **P < 0.01. **(D)** The accumulation of PVY CP protein in tobacco leaves of PVY + H and PVY + Fe groups determined by western blot assays at 1, 3, 5, 7, and 9 dpi.

By observing the symptoms of tobacco plants infected with PVY in four different treatments (P + H, P + Fe, PVY + H, PVY + Fe) at five time points, the results revealed that the tobacco stems began to show brown necrosis at 7 dpi after PVY + H treatment, while did not show obvious disease symptoms treated by PVY + Fe (**Figure 1A**). At 9 dpi, we found that tobacco plants were dwarfed with necrotic veins and yellow leaves, while in PVY + Fe, the stems and veins of the tobacco showed mild brown necrosis (**Figure 1B**). RT-qPCR results showed that the accumulation of PVY RNAs in PVY + Fe treated plants was reduced by 28% at 7 dpi and 68% at 5 dpi, compared with that in PVY + H treated plants (**Figure 1C**). The results of western blot validated that the accumulation of PVY CP in plants after each treatment was generally consistent with the results of RT-qPCR (**Figure 1D**). These results generally proved that Fe had an inhibitory effect on PVY infection in tobacco.

### 3.2 PacBio SMRT sequencing analysis

**Figure 2:**
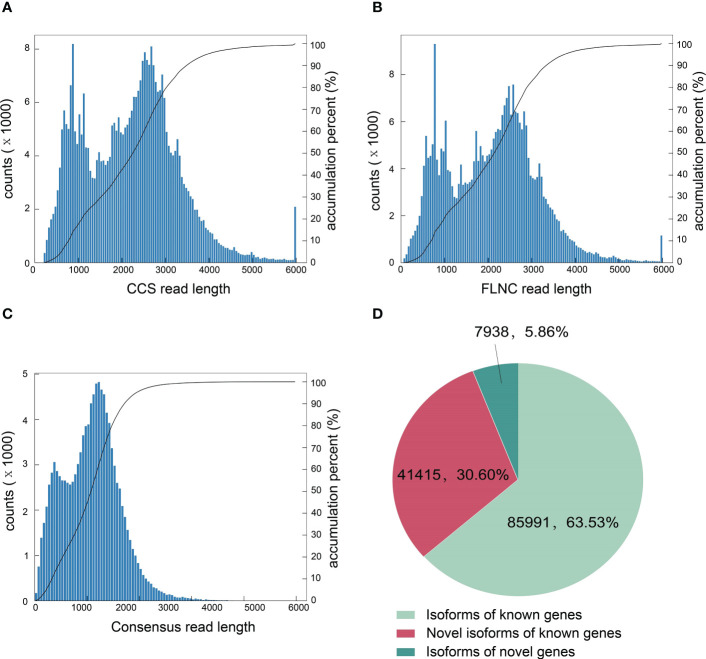
Characterization of tobacco full-length transcripts by PacBio SMRT sequencing. **(A)** The number of CCS reads in different lengths. **(B)** The number of FLNC reads in different lengths. **(C)** The number of consensus reads in different lengths. **(D)** Number and categories of isoforms based on the PacBio platform.

To accurately obtain full-length transcripts and splice variants, the PacBio SMRT sequencing was performed using the pooled sample from all treatments at 1, 3 and 9 dpi. A total of 27.63Gb clean data was obtained with 353,258 circular consensuses (CCS) reads, 316,181 full-lengths non-chimeric (FLNC) sequences and 113,580 high-quality consensus sequences (**Figures 2A–C**). Totally 85,991 isoforms of known genes, 41,415 novel isoforms of known genes and 7,938 isoforms of novel genes were obtained by aligning the transcripts to the *N. tabacum* genome database (**Figure 2D**). The novel transcript sequences obtained were searched for NR, Swissprot, GO, COG, KOG, Pfam, and KEGG databases, and the functional annotations of 39,301 novel transcripts were listed (**Supplementary Table 2**).

### 3.3 Analyses of alternative splicing, alternative polyadenylation and long non-coding RNA

**Figure 3:**
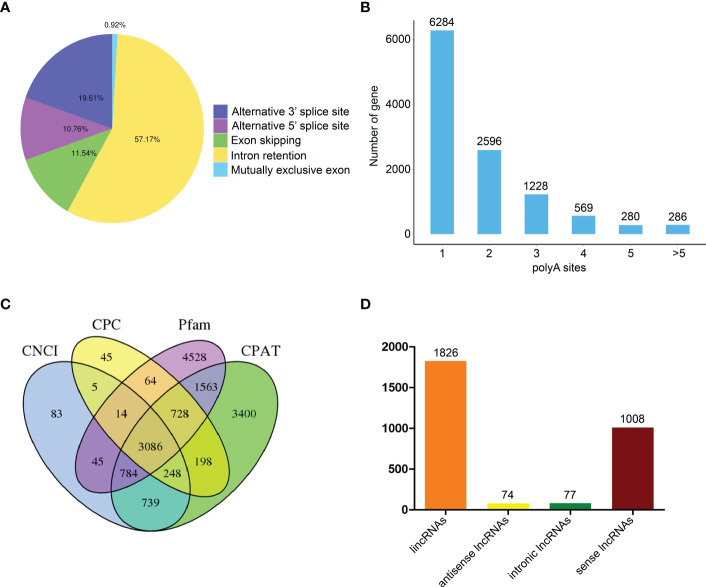
Identification of alternative splicing (AS) events, alternative polyadenylation (APA) and long non-coding RNAs (lncRNAs) based on full-length transcriptome analyses. **(A)** Number and categories of AS events. **(B)** Number of genes with different APA sites. **(C)** Number of lncRNAs based on four analysis methods. **(D)** Number of various lncRNAs.

Alternative splicing (AS) events were subsequently analyzed through an AStalavista tool (Foissac and Sammeth, 2007). A total of 18,074 AS events were indicated in transcripts, including 167 mutually exclusive exon, 10,333 intron retention, 2,086 exon skipping, 1,944 alternative 5’ splice site, and 3,544 alternative 3’ splice site (**Figure 3A**). Moreover, we further analyzed FLNC through TAPIS pipeline to identify alternative polyadenylation (APA) (Abdel-Ghany et al., 2016). We found that 6,284 genes with one APA site, 2,596 genes with two APA sites, 1,228 genes with three APA sites, 569 genes with four APA sites, 280 genes with five APA sites and 286 genes with more than five APA sites (**Figure 3B**). Moreover, a total of 3,086 lncRNAs were identified by coding potential calculator (CPC), coding-non-coding index (CNCI), coding potential assessment tool (CPAT) and Pfam protein domain analyses (**Figure 3C**) (Kong et al., 2007; Sun et al., 2013; Wang et al., 2013). According to the positions of lncRNAs on the reference genome, 1,836 intergenic lncRNAs (lincRNAs), 74 antisense lncRNAs, 77 intronic lncRNAs and 1,008 sense lncRNAs were obtained (**Figure 3D**). The target genes of all lncRNAs were predicted and the results were shown in **Supplementary Table 3**.

### 3.4 Illumina RNA sequencing analysis

To analyze the molecular mechanism of Fe-mediated *N. tabacum* resistance to PVY infection, RNA-Seq was performed on the leaves under four different treatments at three time points. A total of 1307.93 Gb of clean reads were obtained from 36 libraries, and each of these libraries contained ≥ 19.32 Gb of data with Q30 quality scores ≥ 91.67% (**Supplementary Table 4**). These reads were mapped uniquely with the ratios from 76.73% to 93.61% for each library (**Supplementary Table 5**).

### 3.5 Analyses of DEGs in different groups

**Figure 4:**
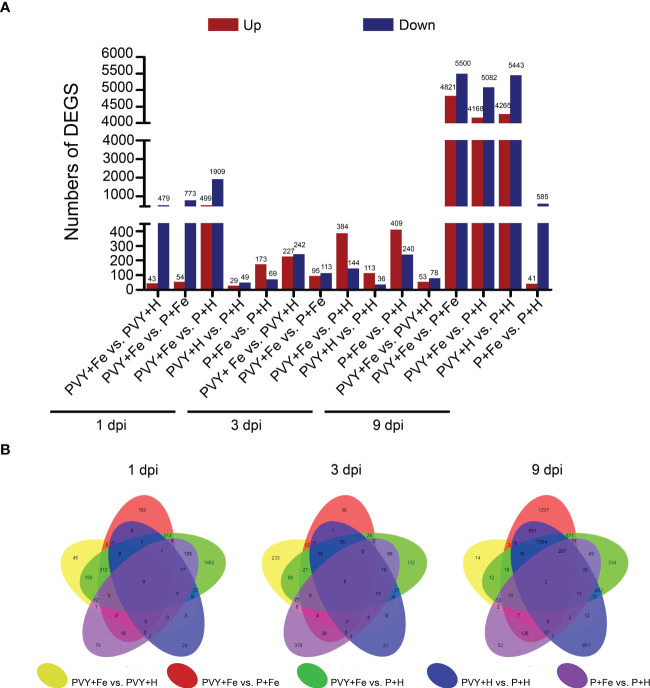
Comparative analyses of DEGs in different treatments at 1, 3, and 9 dpi. **(A)** The number of DEGs in five pairwise comparisons at 1, 3, and 9 dpi. **(B)** The overlapping DEGs in five pairwise comparisons at 1, 3, and 9 dpi showed by Venn diagram.

To identify the genes in response to Fe application under PVY infection, five different pairwise comparisons were conducted (PVY + Fe vs. PVY + H, PVY + Fe vs. P + Fe, PVY + Fe vs. P + H, PVY + H vs. P + H, and P + Fe vs. P + H) at 1, 3, and 9 dpi. Through these comparisons, a total of 2,745 DEGs at 1 dpi, 1,277 DEGs at 3 dpi, and 12,255 DEGs at 9 dpi were obtained (**Figure 4A**). In PVY + H vs. P + H, we found more DEGs at 9 dpi than that at 1 dpi and 3 dpi (**Figures 4A, B**). In PVY + Fe vs. PVY + H, more DEGs were obtained at 1 dpi and 3 dpi than that at 9 dpi (**Figures 4A, B**). These results indicated that the changes in *N. tabacum* gene expression were increased with the progress of PVY infection, and Fe-regulated host resistance to PVY infection mainly functioned at early stage.

### 3.6 GO and KEGG enrichment analyses of DEGs

**Figure 5:**
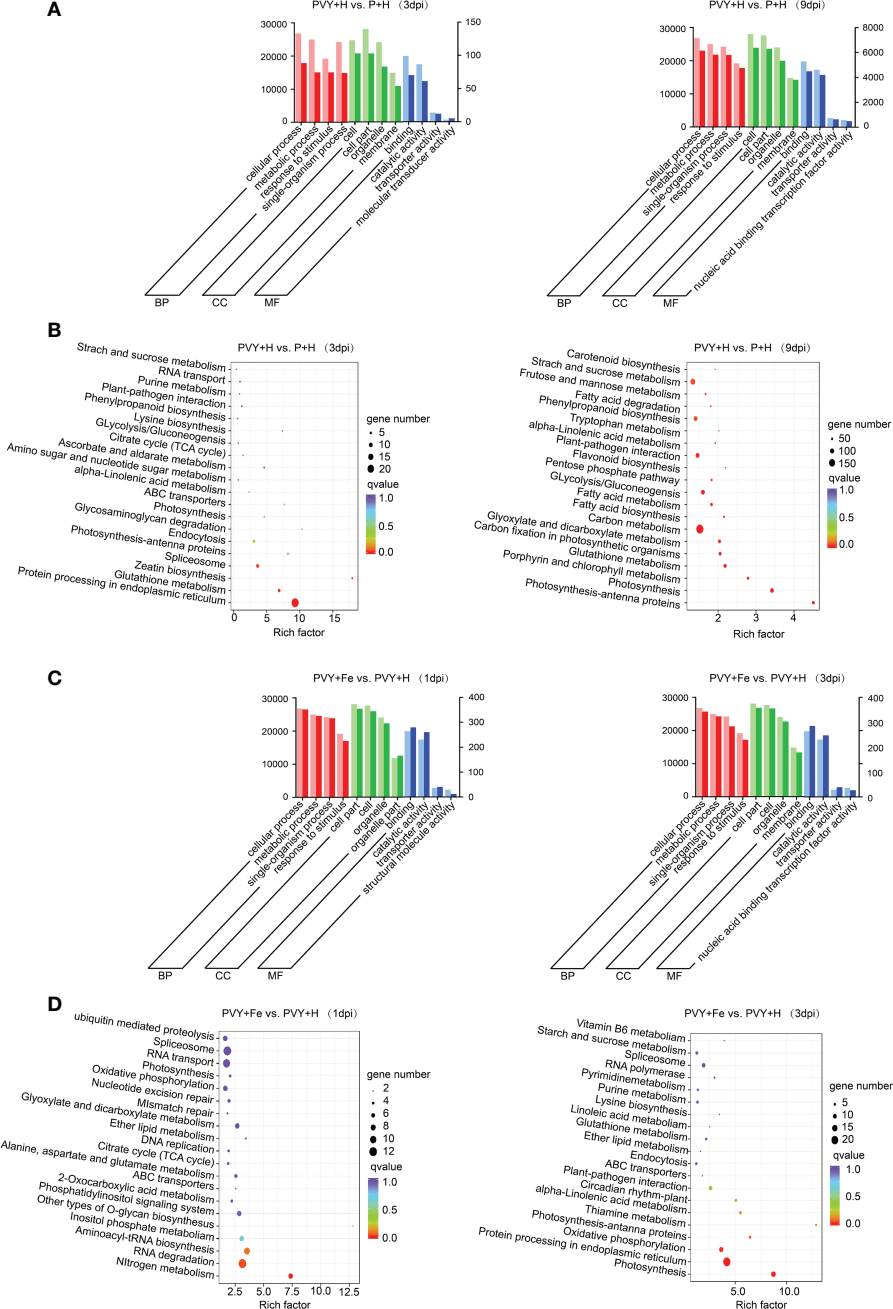
GO and KEGG enrichment analyses of DEGs. **(A)** GO analyses of DEGs in PVY + H vs. P + H at 3 and 9 dpi. **(B)** KEGG enrichment analyses of DEGs in PVY + H vs. P + H at 3 and 9 dpi. **(C)** GO analyses of DEGs in PVY + Fe vs. PVY + H at 1 and 3 dpi. **(D)** KEGG enrichment analyses of DEGs in PVY + Fe vs. PVY + H at 1 and 3 dpi.

To further explore the effects of PVY infection on tobacco biological processes, we analyzed the DEGs in PVY + H vs. P + H at 3 and 9 dpi using GO terms and KEGG pathway enrichment. Through GO analysis, these DEGs were mainly enriched in biological process (BP) terms ‘cellular process’, ‘metabolic process’, ‘response to stimulus’ and ‘single-organism process’, in cellular component (CC) terms ‘cell’, ‘cell part’, ‘organelle’ and ‘membrane’ and in molecular function (MF) terms ‘binding’, ‘catalytic activity’, ‘transporter activity’ and ‘molecular transducer activity’ at 3 dpi (**Figure 5A** and **Supplementary Table 6**). At 9 dpi, the enriched GO terms in BP and CC were the same as those at 3 dpi, while that in MF mainly were ‘binding’, ‘catalytic activity’, ‘transporter activity’ and ‘nucleic acid binding transcription factor activity’ (**Figure 5A** and **Supplementary Table 6**). The results of KEGG enrichment analyses showed that the DEGs were mainly enriched in ‘protein processing in endoplasmic reticulum’, ‘glycosaminoglycan degradation’, ‘zeatin biosynthesis’ at 3 dpi, while in ‘photosynthesis-antenna proteins’, ‘photosynthesis’ and ‘porphyrin and chlorophyII metabolism’ at 9 dpi (**Figure 5B**). To further investigate the responses in tobacco to Fe application during PVY infection, the DEGs obtained in PVY + Fe vs. PVY + H at 1 dpi and 3 dpi were analyzed through GO and KEGG enrichment. The results showed that the DEGs were mainly enriched in the GO terms under the ‘cellular process’, ‘metabolic process’, ‘single-organism process’ and ‘response to stimulus’ of BP, ‘cell part’, ‘cell’, ‘organelle’ and ‘organelle part’ of CC, ‘binding’, ‘catalytic activity’, ‘transporter activity’ and ‘structural molecule activity’ of MF at 1 dpi (**Figure 5C** and **Supplementary Table 6**). At 3 dpi, these DEGs were mainly enriched in BP terms ‘cellular process’, ‘metabolic process’, ‘single-organism process’ and ‘response to stimulus’, CC terms ‘cell part’, ‘cell’, ‘organelle’ and ‘membrane’, and MF terms ‘binding’, ‘catalytic activity’, ‘transporter activity’ and ‘nucleic acid binding transcription factor activity’ (**Figure 5B** and **Supplementary Table 6**). The KEGG enrichment analysis results indicated that the DEGs were mainly in the ‘nitrogen metabolism’, ‘RNA degradation’, ‘aminoacyl-tRNA biosynthesis’ and ‘inositol phosphate metabolism’ at 1 dpi, while in ‘photosynthesis’, ‘protein processing in endoplasmic reticulum’, ‘thiamine metabolism’ and ‘photosynthesis-antenna proteins’ at 3 dpi (**Figure 5D**). These results indicated that PVY infection mainly affected the protein processing, secondary metabolism and biosynthesis of tobacco at 3 dpi, and the photosynthesis of tobacco at 9 dpi. Meanwhile, we found that Fe application exhibited major regulatory effects on protein processing, photosynthesis, oxidative phosphorylation of tobacco at 3 dpi.

### 3.7 Coexpression network analyses of DEGs

**Figure 6:**
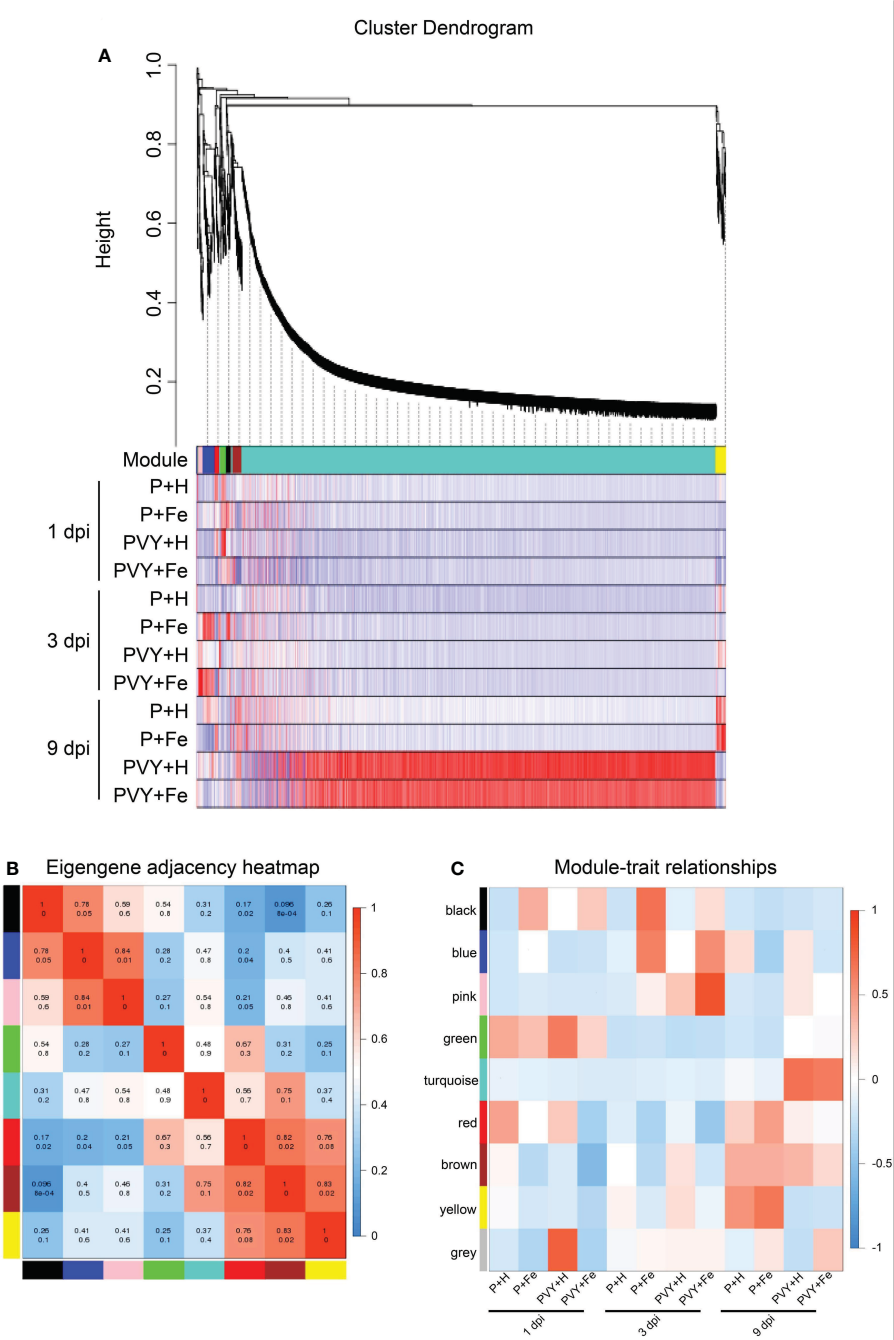
Weighted correlation network analysis (WGCNA) of DEGs. **(A)** Hierarchical cluster tree and heatmap of all DEGs. The hierarchical cluster tree shows co-expression modules identified through WGCNA. Each leaf in the tree represents one DEG. The major tree branches constitute eight modules labeled with different colors. The heatmap shows the relative expressions of the whole DEGs in different modules. **(B)** Eigengene adjacency heatmap of the eight modules shows the correlations among different modules. The darker red represents a higher correlation. The numbers in individual cells represent the correlations. **(C)** Associations between modules and traits. The colors of the modules are the same as that shown in **(A, B)**.

In order to further clarify the roles of Fe application in regulating tobacco anti-PVY infection, WGCNA analysis was performed using all DEGs under different treatments, and eight gene regulatory network modules were obtained, including black module (32 DEGs), blue module (94 DEGs), pink module (33 DEGs), green module (40 DEGs), turquoise module (3,585 DEGs), red module (36 DEGs), brown module (51 DEGs) and yellow module (71 DEGs) (**Figure 6A** and **Supplementary Table 7**). The correlations of modules and modules, modules and different treatments were also analyzed (**Figures 6B, C**). The results showed that there was a strong correlation between turquoise module and brown module. At 9 dpi, these two modules included DEGs that were highly expressed in PVY + H and PVY + Fe (**Figure 6C**). Top GO and KEGG pathway analyses showed that these DEGs were mainly enriched in fatty acid metabolism, fatty acid biosynthesis, plant-pathogen interaction, protein processing in endoplasmic reticulum and photosynthesis (**Supplementary Tables 8**, **9**). These two modules mainly included DEGs related to TFs (*NtbHLH*, *NtMYB*, *NtNAC*, *NtWRKY*), chlorophyll a-b binding protein (*NtCabs*), lipid metabolism (*NtnsLTP*, *NtFAD*, *NtPNPLA*), and molecular chaperones (*NtHSP*, *NtDnaJ*) (**Supplementary Table 8**, **9**). At 3 dpi, the blue and pink modules were highly correlated with PVY + Fe (**Figure 6C**). These DEGs were mainly enriched in plant-pathogen interaction, fatty acid elongation, protein processing in endoplasmic reticulum, glutathione metabolism and plant hormone signal transduction (**Supplementary Table 8**, **9**). These two modules mainly included DEGs related to ethylene signal transduction (*NtERFs*), TFs (*NtWRKYs*), ROS scavenging (*NtGST*, *NtSOD*), protein degradation (*NtE3*), and molecular chaperones (*NtHSP*, *NtDnaJ*) (**Supplementary Tables 8**, **9**).

### 3.8 Analyses of DEGs in different pathways

**Figure 7:**
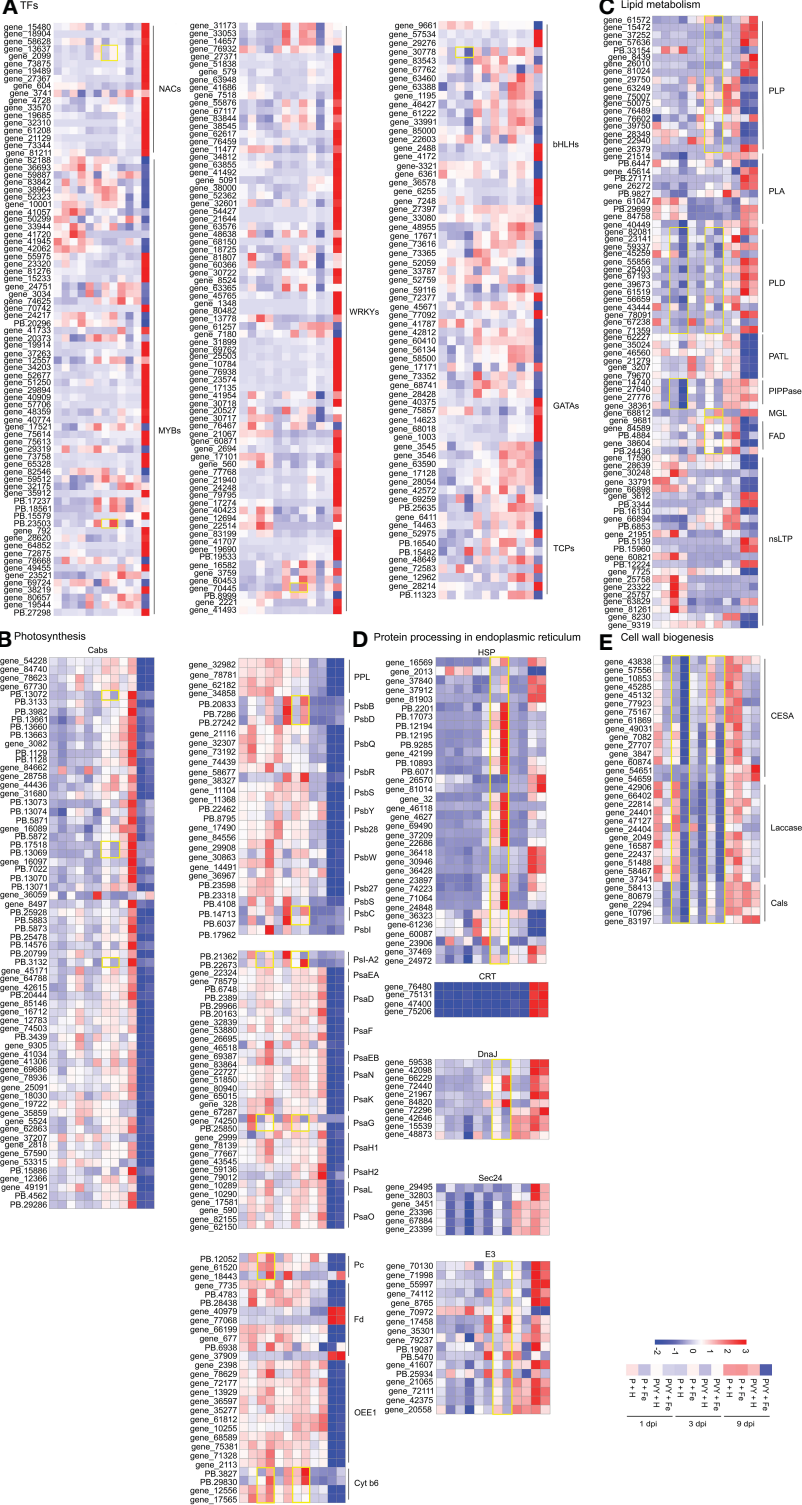
Heat map of the expression levels of DEGs involved in different pathways in tobacco. **(A)** DEGs in transcription factors. **(B)** DEGs in photosynthesis. **(C)** DEGs in lipid metabolism. **(D)** DEGs in protein processing in endoplasmic reticulum. **(E)** DEGs in cell wall biogenesis.

Transcription factors (TFs) play important roles in the regulation of plant life activities (Dubos et al., 2010). In this study, 222 DEGs related to TFs were identified (**Figure 7A** and **Supplementary Table 10**). At 9 dpi, 103 DEGs were up-regulated, including 52 *NtWRKYs* (*NtWRKY15*, *NtWRKY2*, *NtWRKY23*, *NtWRKY26*, *NtWRKY31*, *NtWRKY38*, *NtWRKY40*, *NtWRKY41*, *NtWRKY42*, *NtWRKY43*, *NtWRKY48*, *NtWRKY50*, *NtWRKY51*, *NtWRKY53*, *NtWRKY57*, *NtWRKY65*, *NtWRKY68*, *NtWRKY70*, *NtWRKY71*, *NtWRKY75*, *NtWRKY11*, *NtWRKY6*), eight *NtbHLHs* (*NtbHLH128*, *NtbHLH55*, *NtbHLH66*, *NtbHLH96*), 16 *NtNACs* (*NtNAC2*, *NtNAC72*, *NtNAC8*, *NtNAC29*), 19 *NtMYBs* (*NtMYB315*, *NtMYB330*, *NtMYB4*, *NtMYB108*, *NtMYB144*, *NtMYB1R1*, *NtMYB24*, *NtMYB3*, *NtMYB48*, *NtMYB57*), five *NtGATAs* (*NtGATA24*, *NtGATA26*) and two *NtTCP4*, while 64 DEGs were down-regulated, including 19 *NtMYBs* (*NtMYBAPL*, *NtMYB1R1*, *NtMYB306*, *NtMYB3R-1*, *NtMYB4*, *NtMYB32*, *NtMYB3*, *NtMYB86*), six *NtWRKYs* (*NtWRKY13*, *NtWRKY49*, *NtWRKY22*, *NtWRKY44*), 21 *NtbHLHs* (*NtbHLH122*, *NtbHLH35*, *NtbHLH48*, *NtbHLH49*, *NtbHLH52*, *NtbHLH63*, *NtbHLH71*, *NtbHLH74*, *NtbHLH78*, *NtbHLH79*, *NtbHLH93*, *NtbHLH96*), 12 *NtGATAs* (*NtGATA11*, *NtGATA12*, *NtGATA18*, *NtGATA4*, *NtGATA5*, *NtGATA8*), 6 *NtTCPs* (*NtTCP12*, *NtTCP14*, *NtTCP4*, *NtTCP7*) in PVY + H vs. P + H. In PVY + Fe vs. PVY + H, we found that two *NtNAC2*, one *NtMYB44* and one *NtWRKY22* were up-regulated at 3 dpi, while at 1 dpi, only one *NtbHLH30* was down-regulated. These results suggested that PVY infection seriously impacted the expression levels of many TFs at 9 dpi, while only several TFs might function in the Fe-mediated anti-PVY responses at 3 dpi.

Virus infection often destroys chloroplasts and affects photosynthesis in plants (Zhao et al., 2016). In this study, we identified 154 photosynthesis-associated DEGs (**Figure 7B** and **Supplementary Table 10**). At 3 dpi, six DEGs were up-regulated in PVY + H vs. P + H, while at 9 dpi, a number of DEGs were down-regulated, including 61 chlorophyll a-b binding protein (*NtCab13*, *NtCab16*, *NtCab21*, *NtCab36*, *NtCab37*, *NtCab4*, *NtCab40*, *NtCab50*, *NtCab6A*, *NtCab7*, *NtCab8*, *NtCab10A*, *NtCabP4*), four PsbP-like protein (*NtPPL*), 12 oxygen-evolving enhancer protein (*NtOEE1*), two cytochrome b6 (*NtCyt-b6*), two plastocyanin (*NtPc*), five ferredoxin (*NtFd*) and 44 DEGs that involved in photosystem I (PSI) and II (PSII) assembly. In PVY + Fe vs. PVY + H, we found that *NtPSI-A2*, *NtPsaG*, *NtPc* and *NtCyt-b6* were up-regulated at 1 dpi. At 3 dpi, *NtCabs* (*NtCab16*, *NtCab40*, *NtCab50*) and *NtPsaG* were down-regulated, while *NtPsbB*, *NtPsbD*, *NtPSI-A2*, *NtPsbC* and *NtCyt-b6* were up-regulated. These results indicated that PVY infection strongly suppressed photosynthesis at 9 dpi, and Fe might induce tobacco resistance to PVY infection by regulating cytochrome, chlorophyll, photosystem I and II assembly.

Besides participating in biofilm composition, lipids also play an important role in regulating plant metabolism in response to stress (Lim et al., 2017). Eighty-one DEGs identified at 1, 3 and 9 dpi were found to associate with lipid metabolism and transport (**Figure 7C** and **Supplementary Table 10**). In phospholipid metabolic pathway, we found that five patatin-like protein genes (*NtPLPs*), three phospholipase A genes (*NtPLAs*), five phospholipase D genes (*NtPLDs*) were up-regulated in PVY + H vs. P + H at 9 dpi, while seven *NtPLPs*, two *NtPLAs*, one *NtPLD* and six patellin genes (*NtPATLs*) were down-regulated. In PVY + Fe vs. PVY + H, three *NtPLDs* and four phosphoinositide phosphatase genes (*NtPIPPases*) were down-regulated at 1 dpi, only one *NtPLD* and one *NtPLP* were down-regulated at 3 dpi. In PVY + H vs. P + H at 9 dpi, monoglyceride lipase-like (*NtMGLs*) related to fatty acid synthesis were all up-regulated, and fatty-acid desaturase (*NtFADs*) related to unsaturated fatty acid synthesis were all down-regulated. Interestingly, similar changes in expression levels of *NtMGLs* and *NtFADs* occurred in PVY + Fe vs. PVY + H at 3 dpi. Twenty-three non-specific lipid-transfer protein (*NtnsLTPs*) associated with lipid transport were identified. The expression levels of most of these genes were changed in PVY + H vs. P + H at 9 dpi. However, their expression was not affected by Fe application under PVY infection.

Protein processing in endoplasmic reticulum is closely related to protein folding, transport and degradation, which is essential for the normal function of proteins (Ito and Takeda, 2012). In PVY + H vs. P + H, 15 small heat shock protein (*NtHSP20s*) and two heat shock protein (*NtHSP90s*) genes were up-regulated at 3 dpi, while the expression levels of other genes involved in protein processing in endoplasmic reticulum remained unchanged. At 9 dpi, the expression levels of calreticulin (*NtCRTs*), DnaJ protein (*NtDnaJs*), most of E3 ubiquitin-protein ligase (*NtE3s*), 17 *NtHSP20s* and six *NtHSP90s* were up-regulated, while two *NtHSP20s* and three *NtHSP90* were down-regulated (**Supplementary Table 10**). In PVY + Fe vs. PVY + H, two *NtHSP70s*, two *NtDnaJs*, three *NtSec24s* and five *NtE3s* were down-regulated at 1 dpi. At 3 dpi, 13 *NtHSP20s*, two *NtHSP90s*, three *NtDnaJs* and six *NtE3s* were up-regulated, and two *NtDnaJs* and three *NtE3s* were down-regulated (**Figure 7D** and **Supplementary Table 10**).

Cell wall is the first defense line in plant against pathogen infection (Bacete et al., 2018). In this study, five cellulose synthase genes (*NtCESAs*) were down-regulated at 9 dpi, while the expression levels of other genes associated with cell walls biogenesis remained unchanged in PVY + H vs. P + H. In PVY + Fe vs. PVY + H, the gene expression of almost all the *NtCESAs*, *NtLaccases* and callose synthase genes (*NtCalSs*) were down-regulated at 1 dpi and 3 dpi (**Figure 7E** and **Supplementary Table 10**).

### 3.9 Validation of RNA-Seq data

**Figure 8:**
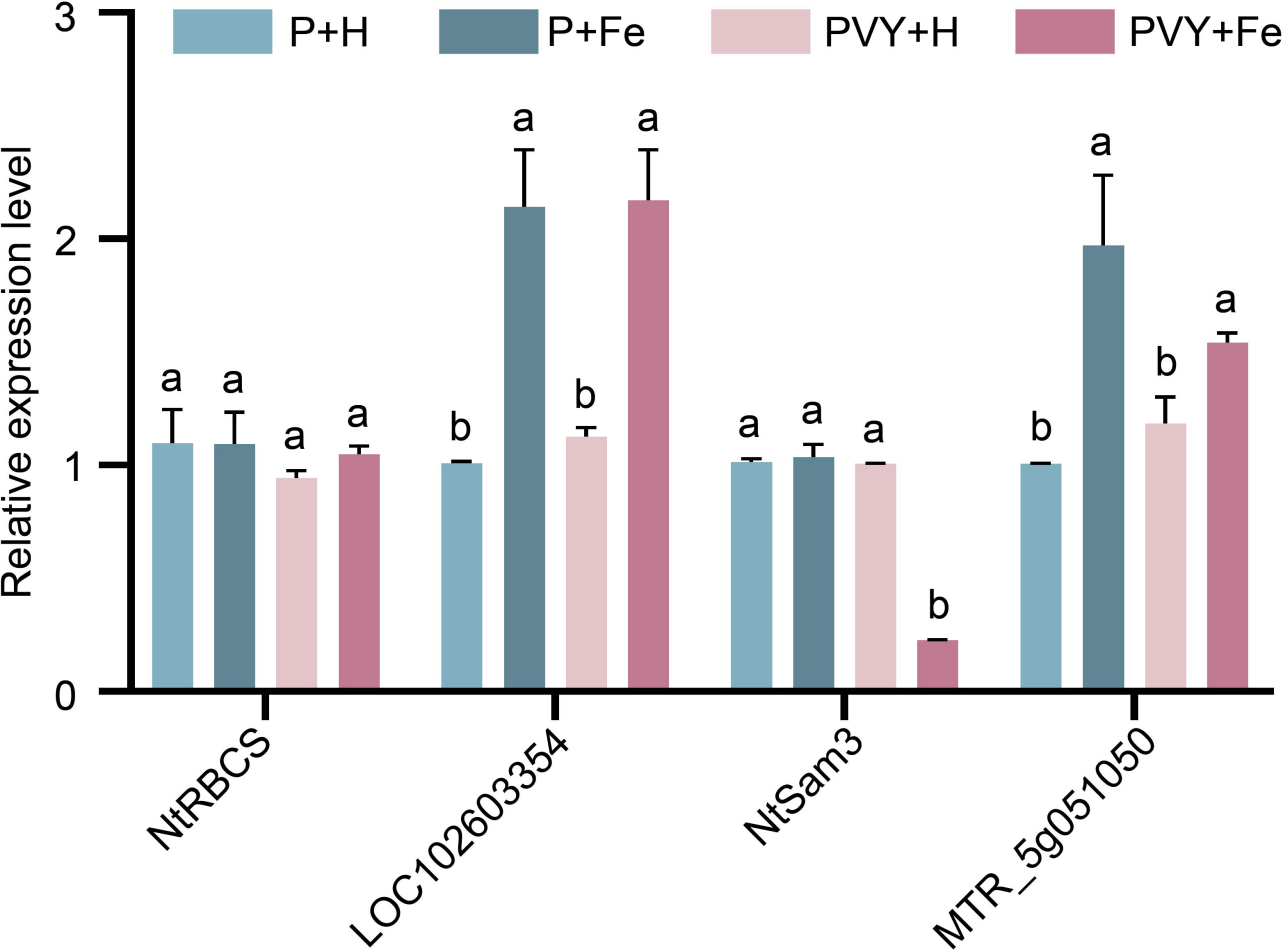
The expression levels of four genes determined by RT-qPCR under different treatments. Different lowercase letters indicate statistical difference between treatments. The statistical significances were determined using one-way analysis of variance followed by Duncan’s multiple comparison test (*P* value < 0.05).

To validate the results of RNA-Seq, four genes were randomly selected to determine their expression levels under different treatments by RT-qPCR (**Figure 8**). The results showed that the expression level of *NtRBCS* (gene_44833) remained unchanged at 1 dpi under four different treatments. At 3 dpi, the results showed that the expression levels of uncharacterized protein *LOC102603354* (PB.15726) and hypothetical protein *MTR_5g051050* (PB.27254) were up-regulated in P + Fe vs. P + H and PVY + Fe vs. PVY + H, while the expression levels of *NtSam3* (gene_42181) were down-regulated in PVY + Fe vs. PVY + H. These results were consistent with those of RNA-Seq.

### 3.10 Functional analyses of tobacco homologous genes in resistance to PVY infection in *Nicotiana benthamiana*

**Figure 9:**
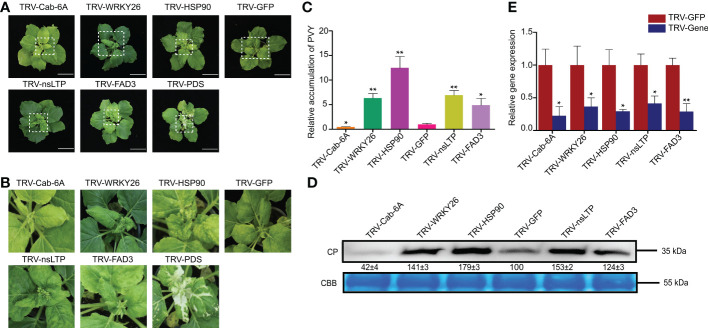
Functional analyses of five homologous genes in resistance to PVY infection through TRV-based VIGS assays in *N. benthamiana*. **(A)** Disease symptoms on different gene-silenced *N. benthamiana* plants after PVY infection. **(B)** Close-up views of upper leaves indicated by white dash boxes in **(A)**. **(C)** The accumulations of PVY genomic RNAs determined by RT-qPCR in different gene-silenced *N. benthamiana*. Asterisks indicate statistical difference between treatments, determined by the two-tailed *t* test (*, *P* < 0.05; **, *P* < 0.01). **(D)** The expression levels of PVY CP proteins in upper leaves of *N. benthamiana*. CP, coat protein; CBB, Coomassie brilliant blue. **(E)** Silencing efficiencies of target genes determined through RT-qPCR. Asterisks indicate statistical difference between treatments, determined by the two-tailed *t* test (*, *P* < 0.05; **, *P* < 0.01).

Transcriptome sequencing analysis showed that *NtCab-6a* was down-regulated at 3 dpi, *NtWRKY26* was up-regulated at 3 and 9 dpi, *NtHSP90* and *NtFAD3* were down-regulated at 9 dpi, and *NtnsLTP* was down-regulated in PVY + Fe vs. PVY + H (**Figure 7**). To further explore the genes in response to Fe application and PVY infection in tobacco, we selected homologs of these five tobacco genes for functional verification through TRV-based VIGS approaches in *N. benthamiana* plants. The results indicated that the leaves of *NbHSP90*-silenced plants showed severe yellowing and mottling compared with controls at 7 days post infiltration, while other gene-silenced plants showed no difference in symptoms. At 10 days post PVY inoculation, the *NbWRKY26*-, *NbHSP90*-, *NbnsLTP*- and *NbFAD3*-silenced plant leaves showed more severe curls and chlorosis than that in the control groups, while the *NbCab-6a*-silenced plant leaves were only slightly curled (**Figures 9A, B**). The accumulations of PVY RNAs and coat proteins were determined respectively by RT-qPCR and western blot, and the results were consistent with the symptomatic observations (**Figures 9C, D**). The gene silencing efficiencies were determined by RT-qPCR, and ranged from 66% to 84% (**Figure 9E**).

## 4 Discussion

Understanding the process of host plants in response to PVY infection can provide a theoretical basis for breeding of new antiviral plant using gene editing. Similarly, the study on nutrient element-induced resistance to PVY infection in host plants is also conducive to improving crop yield from the perspective of PVY control. Our laboratory has reported that leaf spraying with boron or growing in boron nutrient solution can relieve the symptoms of blood flesh disease caused by CGMMV infection in watermelon (Bi et al., 2022a). CGMMV infection affects the metabolism of carbohydrates in watermelon, and exogenous boron induces *ClNIP5;1* and *ClSWEET4* expression, restores and maintains their homeostasis (Bi et al., 2022a). In addition, boron can also induce watermelon resistance to CGMMV infection by regulating ROS-related genes, such as *ClCat*, *ClPrx* and *ClGST* (Guo et al., 2022). It has been reported that zinc treatment on tobacco leaves can improve the host resistance to TMV infection by regulating the expression of *ERF5*, which is involved in the inositol phosphate metabolism (Wang et al., 2022). Copper regulates the protein level of SQUAMOSA promoter-binding-like protein 9 (SPL9) to inhibit transcriptional activation of *MIR528*, resulting in broad-spectrum resistance in *Oryza sativa* (Yao et al., 2022). In this study, the effect of PVY infection in *N. tabacum* gene expression and the molecular basis of Fe application against PVY infection were better understand through PacBio SMRT and Illumina RNA sequencing at 1, 3 and 9 dpi. Through transcriptome analyses, we found that PVY infection mainly affected the expression levels of genes related to photosynthesis and most biological processes, such as lipid metabolism and protein processing (**Figure 5**). The results suggested that Fe application can improve the resistance of tobacco to PVY infection mainly by regulating the expression levels of genes associated with photosynthesis, lipid metabolism, protein processing, cell wall biogenesis and TFs related to disease resistance at early stage of infection.

TFs can regulate the expression of plant genes and play important roles in response to stresses (Amorim et al., 2017). In this study, a total of 227 differentially expressed TFs from six families (NAC, WRKY, MYB, bHLH, GATA and TCP) were identified by RNA-Seq (**Figure 7**), which have been reported to play pivotal roles in plant immunity (Lopez et al., 2015, Behringer and Schwechheimer, 2015). At 1 dpi, we found that *NtbHLH30* and *NtGATA8* were down-regulated, while *NtNAC2*, *NtMYB4* and *NtWRKY22* were up-regulated at 3 dpi in PVY + Fe vs. PVY + H (**Figure 7**). Previously, it has been demonstrated that overexpression of *NbMYB4L* in *N. benthamiana* induced significant resistance to TMV (Zhu et al., 2022). It was demonstrated that *PlWRKY65* in *Paeonia lactiflora* leaves enhanced resistance to *A. tenuissima* by inducing pathogenesis-related (PR) gene expression and increasing jasmonic acid (JA) content (Wang et al., 2020). The CP protein of turnip crinkle virus (TCV) can interact with TIP, a NAC transcription factor, in *Arabidopsis* to inhibit the salicylic acid pathway and promote virus infection (Donze et al., 2014). Therefore, we hypothesized that Fe application could induce the expression of disease-resistant genes by regulating the expression of these above TFs, thereby enhancing tobacco resistance to PVY infection. In addition, silencing of *NbWRKY26* (the homologous gene of *NtWRKY22*) enhanced the accumulation of PVY in *N. benthamiana*, indicating the potential roles of *NbWRKY26* in resistance to PVY infection.

Photosynthesis is the basis of plant life activities and provides energy for all life processes (Zhao et al., 2016). Proteomic analysis of potatoes infected with PVY showed that the viral infection significantly affects the expression of chlorophyll a-b binding protein, as well as plant photosynthesis (Stare et al., 2017). In this study, we found that the expression levels of a large variety of photosynthesis related genes were down-regulated after PVY infection at 9 dpi (**Figure 7**). At 1 dpi, we found that *NtCyt b6*, *NtPSI-A2*, *NtPsaG* and *NtPc* were increased in PVY + Fe vs. PVY + H, while *NtCabs* were down-regulated at 3 dpi. Moreover, the roles of *NbCab-6A* in resistance to PVY infection were verified by TRV-based VIGS, and the results showed that the *NbCab-6A*-silenced plants showed higher PVY resistance. It has been reported that strawberry vein banding virus (SVBV)-encoded P1 can interact with chlorophyll a/b-binding protein of light-harvesting complex II type 1 like (LHC II-1L) and overexpression of *LHC II-1L* can accelerate SVBV infection (Xu et al., 2022). Therefore, *NbCab-6A* promoted PVY infection possibly by directly or indirectly interacting with PVY-encoded proteins, which needs to be further investigated.

There are many kinds of lipids in plants, which are mainly related to membrane structure composition (Lim et al., 2017). Multiple intermediates (i. e. PLD, PI-PLC) in lipid metabolism have been reported to participate in various stress responses by regulating JA and SA signal and inducing host PAMP-triggered immunity (PTI) responses (Lim et al., 2017). In this study, the accumulations of *NtPLD*, *NtPIPPase* and *NtnsLTP* at 1 dpi and *NtPLA*, *NtPLD* and *NtFAD* at 3 dpi were down-regulated in PVY + Fe vs. PVY + H (**Figure 7**). Studies have shown that overexpression of *StLTP6* in potato inhibit the expression of genes involved in RNA silencing pathway and promote the infection of PVY and potato virus S (PVS) (Shang et al., 2022). *StLTP10*-overexpressing improve the expression of disease-resistant genes, and reduce oxidative stress to enhance the resistance to *Phytophthora infestans* (Wang et al., 2021). Fatty acid desaturation (FAD) catalyzes the formation of unsaturated fatty acids (UFA), which is a precursor for the biosynthesis of various hormones, such as JA, SA and terpenes (Xiao et al., 2022). The *fad3fad7fad8* triple mutant of *Arabidopsis* impaired SA synthesis, resulting in an enhanced sensitivity to oomycetes (Browse, 2009). In this study, we found that silencing of *NbnsLTP* or *NbFAD3* in *N. benthamiana* enhanced susceptibility to PVY infection (**Figure 9**). These results indicated that *NbFAD* and *NbnsLTP* play important roles in resistance to PVY infection, which may be related to their regulation on hormone signal transduction and oxidative stress response.

Protein folding and modification in plants occur mainly in the endoplasmic reticulum (Braakman and Hebert, 2013). Molecular chaperones, such as HSP and DnaJ, assist in protein folding in the plant endoplasmic reticulum, (Voellmy and Boellmann, 2007). Calreticulin (CRT) is a soluble protein of the endoplasmic reticulum lumen that recruits other molecular chaperones to facilitate glycosylated protein folding (Caramelo and Parodi, 2008). E3 ubiquitin ligase mainly degrades misfolded proteins (Al-Saharin et al., 2022). In this research, our results indicated that *NtHSP90*, *NtHSP20*, *NtDnaJ* and *NtCRT* were up-regulated at 9 dpi in PVY + H vs. P + H. Interestingly, *NtHSP90*, *NtHSP20* and *NtE3* were also up-regulated at 3 dpi in PVY + Fe vs. PVY + H (**Figure 7**). Through VIGS assays, we found that the accumulation of PVY RNAs and CP proteins were higher in the *NbHSP90*-silenced plants compared with that in the control plants. Previously, many studies have demonstrated that silencing of *NbHSP90* in *N. benthamiana* will lead to the reduction of *PR* gene expression level and promote the infection of potato virus X (PVX), *P. syringae* and TMV (Sangster and Queitsch, 2005). In contrast, a recent study indicated that silencing the homologous gene of potato *StHSP90.5* in *N. benthamiana* reduced PVY accumulation and induced defense-related gene expression (Li et al., 2022). Silencing *NbHSP90* gene in *N. benthamiana* could inhibit *Ralstonia solanacearum* infection by inducing *PR* gene expression (Ito et al., 2015). We hypothesized that different *NbHSP90s* of *N. benthamiana* might have diverse functions, and their roles in antiviral activity against PVY need to be further investigated.

The lignification and callose deposition of plant cell walls prevent pathogen infection and induce resistance responses by transmitting signaling molecules (Bacete et al., 2018). In this study, we found that *NtCESAs*, *NtLaccases* and *NtCaISs* involved in lignification and callose deposition were down-regulated at 9 dpi in PVY + H vs. P + H. The expression level of all *NtCESAs*, *NtLaccases* and *NtCaISs* were down-regulated at 1 and 3 dpi in PVY + Fe vs. PVY + H (**Figure 7**). Cellulose synthase is involved in cellulose synthesis during cell wall development in plants (Bashline et al., 2014). When *Botrytis cinerea* infected *Arabidopsis*, the expression level of cellulose synthase gene was down-regulated to enhanced its disease resistance, which may activate the immune response through signal transduction (Ramírez et al., 2011). Plant laccase regulates cell wall synthesis by participating in lignin synthesis (Wang et al., 2019). Inhibition of *GhLac1* gene expression in cotton can lead to accumulation of JA and secondary metabolites, and improve the resistance to *Verticillium dahlia* (Hu et al., 2018). Callose deposition is a part of the innate immune response of plants and callose is synthesized by callose synthase (Ostergaard et al., 2002). Silencing *CalS1* gene in citrus promoted the infection of *Xanthomonas citri* subsp. *citri* (Enrique et al., 2011). Callose deposition is closely related to abscisic acid content, which is inhibited by low abscisic acid content in *Arabidopsis* (Flors et al., 2008). In conclusion, we speculated that Fe enhanced host resistance to PVY infection possibly by inhibiting the expression of *NtCESA* and *NtLaccase* genes, thus promoting intracellular flow of cell wall signals, activating immune signal transduction, and inducing accumulation of secondary metabolites associated with resistance to disease.

## Data availability statement

The datasets presented in this study can be found in online repositories. The names of the repository/repositories and accession number(s) can be found below: https://www.ncbi.nlm.nih.gov/, PRJNA903693.

## Author contributions

ZX and YW conceived the research project; RL and XL completed element spray tests. CX, ZW, and MA completed transcriptome sequencing tests; CX, HG, YZ, QX, DZ, and QM performed transcriptome data analysis and gene function validation; CX and HG wrote the original draft; MA and ZW revised the manuscript; ZX and YW edited the final manuscript. All authors contributed to the article and approved the submitted version.
